# A new cure model that corrects for increased risk of non-cancer death: analysis of reliability and robustness, and application to real-life data

**DOI:** 10.1186/s12874-023-01876-x

**Published:** 2023-03-25

**Authors:** Laura Botta, Juste Goungounga, Riccardo Capocaccia, Gaelle Romain, Marc Colonna, Gemma Gatta, Olayidé Boussari, Valérie Jooste

**Affiliations:** 1 Evaluative Epidemiology Unit, Department of Epidemiology and Data Science, Fondazione IRCCS “Istituto nazionale dei Tumori”, Via Venezian 1, 20133 Milan, Italy; 2 Registre Bourguignon des Cancers Digestifs, Dijon-Bourgogne University Hospital, F-21000 Dijon, France; 3 UMR 1231, EPICAD team, INSERM, Université Bourgogne-Franche-Comté, Dijon, France; 4 Univ Rennes, EHESP, CNRS, Inserm, Arènes-UMR 6051, RSMS-U 1309, F-3500 Rennes, France; 5 Editorial Board, Epidemiologia e Prevenzione, Milan, Italy; 6 Isere Cancer Registry, Centre Hospitalier Universitaire Grenoble-Alpes, 38043 Grenoble Cedex 9, France; 7 FRANCIM, 1, Avenue Irène Joliot Curie, F-31059 Toulouse, France; 8 Fédération Francophone de Cancérologie Digestive (FFCD), Département de Méthodologie, F-21000 Dijon, France

**Keywords:** Cure model, Increased non-cancer mortality, Population-based data, Robustness, Reliability, Life tables, Net survival, Cancer

## Abstract

**Background:**

Non-cancer mortality in cancer patients may be higher than overall mortality in the general population due to a combination of factors, such as long-term adverse effects of treatments, and genetic, environmental or lifestyle-related factors. If so, conventional indicators may underestimate net survival and cure fraction. Our aim was to propose and evaluate a mixture cure survival model that takes into account the increased risk of non-cancer death for cancer patients.

**Methods:**

We assessed the performance of a corrected mixture cure survival model derived from a conventional mixture cure model to estimate the cure fraction, the survival of uncured patients, and the increased risk of non-cancer death in two settings of net survival estimation, grouped life-table data and individual patients’ data. We measured the model’s performance in terms of bias, standard deviation of the estimates and coverage rate, using an extensive simulation study. This study included reliability assessments through violation of some of the model’s assumptions. We also applied the models to colon cancer data from the FRANCIM network.

**Results:**

When the assumptions were satisfied, the corrected cure model provided unbiased estimates of parameters expressing the increased risk of non-cancer death, the cure fraction, and net survival in uncured patients. No major difference was found when the model was applied to individual or grouped data. The absolute bias was < 1% for all parameters, while coverage ranged from 89 to 97%. When some of the assumptions were violated, parameter estimates appeared more robust when obtained from grouped than from individual data. As expected, the uncorrected cure model performed poorly and underestimated net survival and cure fractions in the simulation study. When applied to colon cancer real-life data, cure fractions estimated using the proposed model were higher than those in the conventional model, e.g. 5% higher in males at age 60 (57% vs. 52%).

**Conclusions:**

The present analysis supports the use of the corrected mixture cure model, with the inclusion of increased risk of non-cancer death for cancer patients to provide better estimates of indicators based on cancer survival. These are important to public health decision-making; they improve patients’ awareness and facilitate their return to normal life.

**Supplementary Information:**

The online version contains supplementary material available at 10.1186/s12874-023-01876-x.

## Background

There is growing awareness that cancer patients, as compared to age- and sex-matched individuals of the general population, may be at an increased risk of death from causes other than the diagnosed cancer, mainly from cardiovascular and respiratory diseases or other independent cancers [1–4]. This increased risk can be partly a consequence of cancer, such as long-term adverse effects of the treatments, and partly due to determinants of cancer, such as a genetic predisposition or environmental and lifestyle related factors. A higher long-term risk of death from other causes in cancer patients than in the general population has been estimated, from Surveillance, Epidemiology, and End Results Program (SEER) cancer registries data, for colorectal, breast and testicular cancer [5]. This increased risk potentially jeopardizes estimations of net survival after cancer for epidemiological and public health purposes.

Net survival is the hypothetical survival that would be measured if the disease under study was the only possible cause of death. It should be used to compare cancer survival in groups with different population mortality [6]. To estimate net survival, two main settings were defined: the cause-specific setting and the relative survival setting, the latter not needing cause of death information. In the relative survival setting, net survival can be estimated through the excess mortality approach, by removing from observed mortality the mortality from other causes, which corresponds to the death that would occur in the cohort in the absence of cancer. Net survival can also be estimated using the ratio approach, by dividing observed survival by the survival that would be observed in the absence of cancer.

Usually, in population-based studies, the mortality (or survival) expected in the absence of cancer is derived from overall mortality (or overall survival) in the general population with the same characteristics. This approximation relies on the commonly accepted assumption that the probability of death by other causes in population-based cohorts of cancer patients is similar to the probability of death by all causes in the general population [6–9]. This assumption might not be true if cancer patients present an increased risk of dying from other causes, related (e.g. adverse effects of treatments) or not (e.g. independent second cancer) with the studied cancer, compared to the general population .

Cure models are used in cancer epidemiology to estimate relative survival under the assumption that a percentage of subjects will not die from cancer (“cured patients”) [10, 11]. This percentage of subjects is materialized by an asymptotic plateau reached by the relative survival curve. In this context, a patients’ increased risk of dying from other causes also impacts mortality rates in those cured, thus challenging the assumption that their mortality rates should be the same as those in the general population. Therefore, popular cancer burden indicators such as relative survival, cure fraction, time-to-cure and survival of uncured patients, can be severely biased if cancer patients exhibit a substantial increased risk of non-cancer death that is not taken into account. Such risk may also affect survival comparisons if it differs among compared populations. Acknowledging this increased risk would have obvious consequences in providing to the patients, parents, clinicians and all the health care stakeholders the estimation of indicators expressing mortality for cancer progression or relapse. For example, it would lead to better targeting of health care programs and enable long-term cancer survivors to obtain credit and insurance more easily. Because the health status of cancer survivors is probably known to the patients themselves, to their physicians and to their insurers, the presence of a comorbid condition that would increase the risk of death is already known and would in any case influence access to loans etc. [12]. The common methods to estimate excess mortality include part of the risk due to comorbidity as well as the excess risk attributed to cancer; they consequently overestimate an individual’s cancer mortality risk.

In the net survival setting, methods have been developed to account for differences between the risk of non-cancer death in cancer patients and the risk of death in the general population, or for insufficiently stratified life tables [13–16]. In the cure modelling setting, a model based on a generalization of mixture cure models [17] has been developed and applied to real-life data (colorectal, breast and lung cancer patients from United States cancer registries) [18]. The reliability of the model and the robustness of its estimates had to be studied in detail before undertaking any extensive applications on real-life data. Once tested, such models could provide practical indications for public health and for the modification of clinical follow-up for long-term survivors and cured cancer patients. This validation task cannot be done using real-life data because cure is an unobserved condition that is treated as a latent variable in cure models. No gold standard is therefore available from real-life data for comparison with model-based cure estimates. In contrast, simulated data, with the generation of large numbers of virtual cohorts with known proportions of cure and precisely defined survival functions, can be useful to test the model’s performance under controlled conditions.

This simulation-based study explored the reliability of a new “corrected” cure model, i.e. including a correction factor to take into account the increased risk of non-cancer death. First, the performance of the model and its statistical properties were explored when all its assumptions were valid. We then analysed the model’s robustness by investigating its performance when some of the underlying assumptions were violated. We also applied the corrected model to real population-based data for colon cancer from the French cancer registries.

## Methods

### Cure models

The proposed corrected mixture cure model can be seen as an extension of the conventional mixture cure model with different assumptions. The latter is used as a reference to assess the performance of the former.

### Conventional mixture cure models

Mixture cure models [19] assume that the cohort of cancer patients is divided into two subgroups: those cured, who will never die from the diagnosed cancer, and the uncured, who will eventually die from the progression or relapse of the disease.

Relative survival (RS) can be estimated for a group of patients (supposed for now homogeneous with respect to age and other possible covariates) by the ratio approach:

$$RS(t)=\frac{S^o(t)}{S^e(t)},$$

where *S*^*o*^(*t*) is the survival observed in the patients’ group and *S*^*e*^(*t*) the survival expected for the same subjects in the absence of cancer and t the time since diagnosis.

Relative survival can also be estimated using the excess hazard approach, assuming that the observed mortality hazard *h*_*O*_(*t*) could be split into two forces of mortality attributable to cancer *h*_*c*_(*t*) and to other causes *h*_*e*_(*t*). This can be written analytically:

$${h}_O(t)={h}_c(t)+{h}_e(t).$$

The relative survival context assumes the expected mortality hazards of patients to be equal to those observed in a general population group comparable for geographic area, calendar year, age and sex, and sometimes for other known characteristics. This implies at the individual level that:

$${h}_e(t)={h}^{\ast }(t)={h}^{\ast}\left( age+t, sex, year+t\right)$$

where age and year are the patient’s age at diagnosis and year of diagnosis.

The cumulative observed hazard can be written as:

$${H}_O(t)={\int}_0^t{h}_c(v) dv+{\int}_0^t{h}^{\ast }(v) dv,$$

And observed survival can be written as:

$${S}_O(t)=\exp \left[-{H}_O(t)\right]=\exp \left[-{\int}_0^t{h}_c(v) dv\right]\exp \left[-{\int}_0^t{h}^{\ast }(v) dv\right],$$

where $\exp \left[-{\int}_0^t{h}_c(v) dv\right]$ corresponds to the relative survival function and $\exp \left[-{\int}_0^t{h}^{\ast }(v) dv\right]$ corresponds to the survival function for the general population.

The conventional mixture survival model expresses relative survival as a mixture of two net survival functions attributed to uncured (*S*_*u*_(***X***, *t*)) and cured patients (*S*_*cured*_(***X***, *t*)), and can be expressed as:

$$RS(t)=\pi \left(\boldsymbol{Z}\right){S}_{cured}\left(\boldsymbol{X},t\right)+\left(1-\pi \left(\boldsymbol{Z}\right)\right){S}_u\left(\boldsymbol{X},t\right)$$

where *S*_*cured*_(***X***, *t*) = 1 and *S*_*u*_(***X***, *t*) can be specified by any parametric survival function. There are a wide range of distribution functions to choose from, and in the mixture model we specified *S*_*u*_(***X***, *t*) as a Weibull function. The Weibull distribution is flexible enough to enable a monotonic increasing or decreasing mortality rate for the uncured group. The parametrization is *S*_*u*_(***X***, *t*) = exp(−*λt*^*γ*^)^exp(***δX***)^, where *λ*> 0 and *γ* > 0 are respectively the scale and shape parameters considered as constant and ***δ*** is the proportional effect of covariates **X** on the baseline survival of uncured patients. To ensure that π remains between 0 and 1, we also specified *π*(***Z***) with a logistic link function allowing a linear effect **β** of covariates **Z** on the cure fraction. Its analytical expression can be written as:

$$\pi \left(\boldsymbol{Z}\right)={\left[1+\exp \left(-{\beta}_0-\boldsymbol{Z}\boldsymbol{\beta } \right)\right]}^{-1}.$$

Other link functions can be used instead of the logistic, for example we used the identity link (π = **Zβ)** [19] for an ancillary analysis addressed in the discussion and presented in the Supplementary material Table 1.

The final expression of relative survival and excess hazard h_c_ in a conventional mixture cure model can be expressed as:

$$RS(t)={\left[1+\exp \left(-{\beta}_0-\boldsymbol{Z}\boldsymbol{\beta } \right)\right]}^{-1}+\left\{1-{\left[1+\exp \left(-{\beta}_0-\boldsymbol{Z}\boldsymbol{\beta } \right)\right]}^{-1}\right\}\kern0.5em \exp {\left(-{\lambda t}^{\gamma}\right)}^{\exp \left(\boldsymbol{X}\boldsymbol{\delta } \right)}$$

And

$${h}_c(t)=\frac{\left\{1-{\left[1+\exp \left(-{\beta}_0-\boldsymbol{Z}\boldsymbol{\beta } \right)\right]}^{-1}\right\}{\gamma \lambda t}^{\gamma}\exp \left(\boldsymbol{X}\boldsymbol{\delta } \right)\exp {\left(-{\lambda t}^{\gamma}\right)}^{\exp \left(\boldsymbol{X}\boldsymbol{\delta } \right)}}{{\left[1+\exp \left(-{\beta}_0-\boldsymbol{Z}\boldsymbol{\beta } \right)\right]}^{-1}+\left\{1-{\left[1+\exp \left(-{\beta}_0-\boldsymbol{Z}\boldsymbol{\beta } \right)\right]}^{-1}\right\}\exp {\left(-{\lambda t}^{\gamma}\right)}^{\exp \left(\boldsymbol{X}\boldsymbol{\delta } \right)}}$$

where **X** is the vector of covariates acting on the survival of the uncured.

### Corrected mixture cure model (Model(1))

Relaxing the comparability assumption usually considered in the excess hazard approach, we set patients’ expected hazard equal to that in the general population multiplied by a constant parameter $\alpha\!:\ h_{e}(t) = ah^{*}(t)=ah^{*}(age+t,sex,year+t),$  with *α* > 0 .

The cumulative observed hazard can be written as:

$${H}_O(t)={\int}_0^t{h}_c(v) dv+{\int}_0^t{\alpha h}^{\ast }(v) dv$$

The proposed excess hazard function is the same as that of the conventional model, but the estimated parameters are different due to the correction of the expected hazard, as in Philips et al. [17].

The observed survival can be written as:

$${S}_O(t)=\exp \left[-{H}_O(t)\right]=\exp \left[-{\int}_0^t{h}_c(v) dv\right]\exp {\left[-{\int}_0^t{h}^{\ast }(v) dv\right]}^{\alpha },$$

where $\exp {\left[-{\int}_0^t{h}^{\ast }(v) dv\right]}^{\alpha }=\kern0.5em {S}^{\ast }{\left(\textrm{t}\right)}^{\alpha}\kern0.5em$ corresponds to survival in the general population corrected by the scale parameter.

The value of parameter α, which is defined on *ℝ*^+^, can be interpreted as a hazard ratio. α > 1 indicates that mortality due to other causes in the cohort under study is higher than that in the general population, α = 1 a null effect, as implicit in the conventional cure models and α < 1 a lower mortality. We assume α to express a fixed effect and to be independent of age.

The final expression of observed survival in the corrected mixture cure model can be expressed as:

$${S}_O(t)={\left[1+\exp \left(-{\beta}_0-\boldsymbol{Z}\boldsymbol{\beta } \right)\right]}^{-1}\ast {S}^{\ast }{\left(\textrm{t}\right)}^{\alpha }+\left\{1-{\left[1+\exp \left(-{\beta}_0-\boldsymbol{Z}\boldsymbol{\beta } \right)\right]}^{-1}\right\}\ast \exp {\left(-{\lambda t}^{\gamma}\right)}^{\exp \left(\boldsymbol{X}\boldsymbol{\delta } \right)}\ast {S}^{\ast }{\left(\textrm{t}\right)}^{\alpha }$$

The corrected model expressed here (from here on called Model(1)), with the constraint α ≡ 1 gives the conventional cure model [19] with the same parameterization of age effects and uncured net survival function.

### Model estimation

Model parameters were estimated using the maximum likelihood method from both individual and grouped data.

As in De Angelis et al. (1999), the total log-likelihood using the individual data approach in the conventional model can be expressed as:

$$l\left(\boldsymbol{\beta}, \gamma, \lambda, \boldsymbol{\delta} \right)=\sum_{j=1}^N-{d}_i\ln \left(\frac{\left(1-\pi \left(\left.{\boldsymbol{Z}}_i\right|\boldsymbol{\beta} \right)\right){f}_u\left(\left.{\textbf{X}}_{i,}{\textrm{t}}_i\right|\boldsymbol{\delta} \right)}{\pi \left(\left.{\boldsymbol{Z}}_{\boldsymbol{i}}\right|\boldsymbol{\beta} \right)+\left(1-\pi \left(\left.{\boldsymbol{Z}}_{\boldsymbol{i}}\right|\boldsymbol{\beta} \right)\right){S}_u\left(\left.{\textbf{X}}_{i,}{\textrm{t}}_i\right|\boldsymbol{\delta} \right)}+{h}^{\ast}\left({\textrm{t}}_i\right)\right)+\ln \Big(\pi \left(\left.{\boldsymbol{Z}}_{\boldsymbol{i}}\right|\boldsymbol{\beta} \right)+\left(1-\pi \left(\left.{\boldsymbol{Z}}_{\boldsymbol{i}}\right|\boldsymbol{\beta} \right)\right){S}_u\left(\left.{\textbf{X}}_{\boldsymbol{i}},{\textrm{t}}_i\right|\boldsymbol{\delta} \right)\Big)+\ln \left({S}^{\ast}\left({\textrm{t}}_i\right)\right)$$

where ***β,****γ*, *λ****,δ*** are the vectors of parameters to be estimated using the maximum likelihood method, t_i,_ d_i_, and h_i_* are, respectively, the time at death or censoring, the censoring index, and the population death hazard for the i-th individual observation among N individuals. **X** and **Z** are the covariates associated with survival of uncured patients and cure fraction, respectively. f_U_(**X**_*i*_, t_*i*_|***δ***) and S_U_(**X**_*i*_, t_*i*_|***δ***) are respectively the density and survival of uncured patients at time t_i_ depending on the effect *δ* of covariates acting on the baseline density or survival and S*(t_i_) is the general population survival at time t_i,_ which is a constant term and can be removed from the likelihood in the conventional model.

Using the same idea as in the conventional model, the total log-likelihood using the individual data in the proposed corrected model can be expressed as:

$$l\left(\boldsymbol{\beta}, \gamma, \lambda, \boldsymbol{\delta}, \alpha \right)=\sum_{i=1}^N-{d}_i\ln \left(\frac{\left(1-\pi \left(\left.{\boldsymbol{Z}}_{\boldsymbol{i}}\right|\boldsymbol{\beta} \right)\right){f}_u\left(\left.{\textbf{X}}_i,{\textrm{t}}_i\right|\boldsymbol{\delta} \right)}{\pi \left(\left.{\boldsymbol{Z}}_i\right|\beta \right)+\left(1-\pi \left(\left.{\boldsymbol{Z}}_{\boldsymbol{i}}\right|\boldsymbol{\beta} \right)\right){S}_u\left(\left.{\textrm{t}}_i\right|\boldsymbol{\delta} \right)}+\alpha {h}^{\ast}\left({\textrm{t}}_i\right)\right)+\ln \left(\pi \left(\left.{\boldsymbol{Z}}_{\boldsymbol{i}}\right|\boldsymbol{\beta} \right)+\left(1-\pi \left(\left.{\boldsymbol{Z}}_{\boldsymbol{i}}\right|\boldsymbol{\beta} \right)\right){S}_u\left(\left.{\textbf{X}}_{\boldsymbol{i}},{\textrm{t}}_i\right|\boldsymbol{\delta} \right)\right)+\ln \left({S}^{\ast }{\left({\textrm{t}}_i\right)}^{\alpha}\right)$$

Notice that in the estimation step the S* cannot be removed as α is a parameter to be estimated, whereas it can in the conventional model.

The estimation from group data was carried out by building relative survival tables, stratified by relevant predictor variables, from each generated sample and from survival data for the general population. In this study we used the commonly used Ederer II relative survival, but other estimators could be considered, particularly in between-population comparative analyses. The binomial log-likelihood for the j-th interval of the life table of k-th strata was derived similarly to the formula provided by Dickman [20]:

$${{\textrm{d}}_{\textrm{jk}}}^{\ast}\log \left[1-{S}_{0jk}\right]+\left({\textrm{l}}_{\textrm{jk}} {\textrm{-d}}_{\textrm{jk}}\right)\ast \log \left[{S}_{0jk}\right]$$

and l_jk_ = (n_jk_ – 0.5w_jk_), the effective number of patients in each j and k combination,

where n_jk_, d_jk_, and w_jk_ were respectively the number of those alive at the start, dead and censored during the interval for k-th strata, and *S*_*Ojk*_ is the j-th interval-specific observed survival for k-th strata.

The log-likelihood using the grouped data approach in the conventional model can be expressed as: d_jk_*log[1- $\left({RS}_{jk}\ast {S}_{jk}^{\ast}\right)\Big]$  + ( l_jk -_ d_jk_)* log[${RS}_{jk}\ast {S}_{jk}^{\ast }$].

Where ${S}_{jk}^{\ast }$ and *RS*_*jk*_ are the expected survival and patients’ relative survival estimates in each j and k combination.

Using the same idea as in the conventional model, the log-likelihood using the grouped data in the proposed model can be expressed as:

$${{\textrm{d}}_{\textrm{jk}}}^{\ast}\log \left[1-\left({RS}_{jk}+\upalpha \log \left({S}_{jk}^{\ast}\right)\right)\right]+\left({\textrm{l}}_{\textrm{jk-} }{\textrm{d}}_{\textrm{jk}}\right)\ast \log \left[{RS}_{jk}+\upalpha \log \left({S}_{jk}^{\ast}\right)\right]$$

Note that, differently from the standard cure models, survival in the population has to be taken into consideration in the maximization of the log likelihood, due to the presence of α.

Using STATA, command *strs* was used to provide Ederer II estimates for grouped data, and command *ml* with the *lnf* method to determine Maximum likelihood estimations, based on the numerical calculation of derivatives, was used to maximize each sample likelihood. The results provided for each sample were stored, summarized and presented as a synthesis.

### Simulation

Virtual samples representing cohorts of patients were built by means of a pseudorandom number-generating algorithm. Each virtual case was independently represented by three variables: age at diagnosis (restricted in all analyses from 40 to 74 years), follow-up time (0–15 years), and censoring index (alive, dead). Age at diagnosis was randomly generated from a uniform distribution within age classes 40–57, 58–64, 65–69, and 70–74, with each class including 25% of all cases.

In the simulations, age at diagnosis was the only covariate associated with both cure fraction (vector **Z**) and survival of uncured patients (vector **X**) from now on reported as x. In model applications, a standardized age variable x = (age-60)/15 was used.

Follow-up time and the censoring index were generated as follows.

- The probabilities of administrative censoring (P_AC_) and of loss to follow-up (P_LF_) were assigned, and the corresponding times to censoring T_C1_ and T_C2_ were sampled from uniform distribution *U[0, 15]* with probability P_AC_ and P_LF_, or set at a maximum (15 years) with probability 1-P_AC_ and 1-P_LF_, respectively. The final censoring time was T_C_ = min(T_C1_, T_C2_).
- A Weibull distribution with parameters λ_P_ (scale) and γ_P_ (shape) was fitted to a set of observed survival data derived from population lifetables. The patients’ expected survival time T_ED_ to causes of death other than cancer was randomly sampled by the inverse transformation method from the estimated Weibull distribution, rising the general population survival probabilities to the power α to simulate the relative risk of non-cancer death of cancer patients.
- The time to cancer death T_CD_ was randomly sampled from a mixture cure model assuming that the net survival of uncured patients followed a Weibull distribution with scale and shape parameters λ_C_ and γ_C_ considered constant and with *δ*, representing the age effect on uncured survival. Sampling u* from the uniform distribution U[0, 1] we set:

$${\textrm{u}}^{\ast }=\uppi \left(\textrm{x}\right)+\left[1-\uppi \left(\textrm{x}\right)\right]{\left[\exp \left(-{\uplambda}_{\textrm{C}}{{\textrm{T}}_{\textrm{CD}}}^{\upgamma_{\textrm{C}}}\right)\right]}^{\exp \left[-\updelta \textrm{x}\right]}$$

Where π(x) = 1/[1 + exp.(−β_0_ -β x)] and β_0_ = ln[π(60) /(1 - π(60))]. The reference age was 60 years, and π(60) and β were fixed according to scenario. If u* > π(x) then we obtained:

$${T}_{CD}={\lambda}_c{\left[-\ln \left(\frac{u^{\ast }-\pi (x)}{1-\pi (x)}\right)\right]}^{{}^{1}\!\left/ \!{}_{{\gamma}_c}\right.}$$

Otherwise, T_CD_ was set to infinity.

- The time to death from diagnosis was then defined as T_D_ = min(T_ED_, T_CD_), and the final time of follow-up by T = min(T_C_, T_D_).
- Finally, the censoring index d was equal to 0 (alive) if T_D_ > T_C_, or equal to 1 (dead) if T_C_ ≥ T_D_.

The whole set of true parameter values considered in the simulation analysis were chosen to mimic the survival pattern of common cancers and the demographic characteristics of real patients’ groups and general populations. For notational simplicity, the symbols π_60_, λ and γ will be used in the following instead of π(60), λ_c_, and γ_c_. The distribution of ages at diagnosis was derived from that observed in (both sexes) incident cases collected by the French network of cancer registries (FRANCIM) [21] during the period 1995–2009 and gathered in the FRANCIM common database. This database also provided the probability of administrative censoring (50%) and of loss to follow-up (3%). The survival distribution parameters in the population, fixed as λ_P_ = 88; γ_P_ = 11, were derived from the French general population life-tables, also for both sexes combined, for the year 2002 [19]. The underlying α in the simulated samples were attributed the values 0.8; 1.0; 1.2; 1.5; 2.0, the first indicating some (perhaps unrealistic) protective effect, the second no effect, and the others an increased risk of non-cancer death in patients as compared with the general population. The underlying true values of parameters determining the proportion of cured patients (π_60_ and β) and survival of the uncured (λ, γ, and δ) were derived from preliminary model applications to real data [18, 21–23], and are shown in Table 1. They were grouped under two scenarios, mirroring the behaviour of lung and breast cancers. In the following, the two scenarios will be named after the corresponding cancer within quotes (“Breast” or “Lung”).

**Table 1:** Performance indicators of Model(1) using Maximum likelihood (ML) estimation by different values of α

Scenario	ML estimation using	True value α	AB						SD						CVR					
			α	π_**60**_	λ	γ	β	δ	α	π_**60**_	λ	γ	β	δ	α	π_**60**_	λ	γ	β	δ
BREAST^a^	grouped	2	−0.005	−0.004	0	0.002	−0.011	− 0.005	0.115	0.043	0.012	0.048	0.199	0.157	94%	93%	93%	95%	93%	94%
	individual	2	0.004	−0.003	0.001	0.002	−0.002	− 0.007	0.11	0.04	0.012	0.041	0.187	0.15	93%	93%	94%	94%	93%	93%
	grouped	1.5	−0.001	−0.003	0	0.002	−0.007	−0.004	0.099	0.038	0.011	0.046	0.184	0.146	94%	93%	95%	95%	94%	94%
	individual	1.5	0.005	−0.002	0.001	0.002	−0.001	− 0.006	0.095	0.036	0.011	0.04	0.172	0.139	94%	93%	95%	95%	93%	94%
	grouped	1.2	0	−0.003	0	0.002	− 0.007	− 0.003	0.09	0.036	0.01	0.045	0.174	0.139	93%	94%	96%	96%	94%	94%
	individual	1.2	0.005	−0.002	0.001	0.002	−0.001	− 0.005	0.086	0.033	0.01	0.039	0.163	0.132	93%	93%	95%	95%	94%	94%
	grouped	1	−0.001	−0.003	0	0.002	−0.009	− 0.001	0.084	0.034	0.01	0.044	0.169	0.134	93%	94%	95%	96%	94%	93%
	individual	1	0.002	−0.002	0	0.002	− 0.003	− 0.004	0.08	0.032	0.01	0.038	0.158	0.127	93%	93%	95%	95%	94%	94%
	grouped	0.8	−0.001	−0.002	0	0.002	−0.007	−0.002	0.077	0.033	0.009	0.043	0.161	0.129	94%	92%	95%	95%	93%	93%
	individual	0.8	0.002	−0.002	0	0.002	−0.002	− 0.004	0.074	0.031	0.009	0.037	0.152	0.123	94%	92%	95%	95%	93%	94%
LUNG^b^	grouped	2	− 0.006	0.002	− 0.009	− 0.001	0.003	0.003	0.206	0.004	0.014	0.015	0.065	0.023	96%	92%	89%	95%	95%	95%
	individual	2	0.005	0	0	0	0.001	−0.001	0.199	0.004	0.012	0.008	0.063	0.022	96%	97%	95%	94%	96%	94%
	grouped	1.5	−0.004	0.002	− 0.009	− 0.001	0.003	0.003	0.165	0.004	0.013	0.014	0.063	0.023	96%	92%	89%	94%	95%	94%
	individual	1.5	0.004	0	0	0	0	0	0.16	0.004	0.012	0.008	0.061	0.022	96%	96%	95%	95%	96%	94%
	grouped	1.2	0	0.002	−0.009	0	0.004	0.003	0.149	0.004	0.013	0.014	0.062	0.023	95%	92%	90%	94%	94%	93%
	individual	1.2	0.006	0	0	0	0.001	0	0.143	0.004	0.012	0.008	0.061	0.022	95%	96%	95%	95%	95%	94%
	grouped	1	0.001	0.002	−0.009	0	0.004	0.003	0.132	0.004	0.013	0.014	0.061	0.023	96%	91%	90%	94%	94%	94%
	individual	1	0.006	0	0	0	0.001	−0.001	0.127	0.004	0.012	0.008	0.06	0.022	95%	95%	95%	95%	95%	94%
	grouped	0.8	−0.002	0.002	− 0.009	0	0.003	0.003	0.121	0.004	0.013	0.014	0.06	0.022	95%	91%	90%	94%	94%	93%
	individual	0.8	0.001	0	0	0	0	0	0.117	0.004	0.012	0.007	0.059	0.022	95%	96%	95%	94%	95%	94%

Model parameters:

α: hazard ratio representing non-cancer death risk of patients versus general population; π_60_: proportion of cured at age 60 at diagnosis; β: rate of increase of proportion of cured per 15 years of age; λ, γ: scale and shape parameters of the survival function of uncured patients; δ: age effect of uncured survival per 15 years. Estimated over 1000 simulation runs; Sample size 10,000; follow-up 15 years

Absolute Bias (AB) = Mean(estimates - true value); Standard Deviation (SD) = standard deviation over the set of 1000 estimates; Coverage (CVR) = the proportion of the time that the estimated 95% confidence intervals contained the true value

^a^True values: π_60_ = 0.7, λ =0.1, γ =1.1, β = − 0.15 and δ =0

^b^True values: π_60_ = 0.1, λ =0.9, γ =0.8, β = − 0.75 and δ = − 0.3

Finally, one thousand samples were generated for each scenario, as defined by a specific set of simulation parameters (α, π_60_, λ, γ, β, δ). Depending on the specific objective, the number of cases generated for each sample varied from a minimum of 500 to a maximum of 20,000.

### Performance indicators

To allow comparison with the results obtained by the conventional cure model some of the estimations were done with the constraint α = 1 and with unconstrained Model(1), thereby taking into account the increased risk of non-cancer death.

From the considered models, we obtained estimates of six parameters: α, π_60_, λ, γ, β, and δ. Intrinsically, the conventional cure model did not estimate α. We indicated as *true values* the values of parameters used to generate the samples, and considered them the gold standard to be compared with model-based estimates. The following performance indicators were calculated for each parameter from the set of 1000 samples generated under a specific scenario.

- Absolute Bias (AB) = Mean(estimates - true value)
- Standard Deviation (SD) = standard deviation over the set of 1000 estimates
- Coverage (CVR) = the proportion of the time that the estimated 95% confidence intervals contained the true value

### Robustness analysis

We investigated the estimates provided by Model(1) when some of the underlying assumptions went against the data. In particular, we analysed the model’s performance in three different situations: the times to cancer death of uncured patients do not follow a Weibull distribution; the increased risk of non-cancer death is dependent on age at diagnosis; the increased risk of non-cancer death varies randomly among patients. All of the robustness analyses were carried out by conducting 1000 independent runs with simulated samples of 10,000 cases each.

#### The times to cancer death of uncured patients do not follow a Weibull distribution

Model(1) assumes that the relative survival of uncured patients diagnosed at the reference age 60 (x = 0) follows the Weibull distribution with parameters λ and γ.

$${\textrm{S}}_{\textrm{u}}\left(\textrm{t},\textrm{x}\right)=\exp {\left(-{\uplambda \textrm{t}}^{\upgamma}\right)}^{\exp \left[-\updelta \left(\textrm{x}\right)\right]}$$

In this analysis, data were generated according to the corrected exponential Weibull distribution [24]:

$${\textrm{S}}_{\textrm{u}}\left(\textrm{t},\textrm{x}\right)={\left\{1-{\left[1-\exp \left(-{\uplambda \textrm{t}}^{\upgamma}\right)\right]}^{\uptheta}\right\}}^{\exp \left[-\updelta \left(\textrm{x}\right)\right]}$$

where the second shape parameter θ modulates the distance from the Weibull. When θ < 1, the hazard is U-shaped, i.e. first decreasing and then increasing. The opposite bell-shaped pattern is obtained when θ > 1. Note that at ages different from 60, x ≠ 0, the survival function (2) no longer follows an exponential Weibull distribution.

Model(1), with uncured survival function specified by (1) was then fitted to data generated from survival function (2). We tested the model under the two scenarios “Breast”, and “Lung“, with θ varying between 0.25 and 4. The shapes of the probability density functions generated under these values are plotted in Supplementary material Figure 1 together with the shape of the corresponding Weibull distributions. The range was considered sufficiently wide to include most of the real-world situations. Note that values of θ far from 1 led to drastic changes in time to death distribution. For instance, 5-year survival from the Weibull distribution (λ =0.4; γ =0.8; θ = 1) increases from 23 to 66% when θ = 4, and decreases to 8% when θ = 0.5. For this reason, actual estimates were compared with underlying values only for parameters α, π, β and δ.

#### The increased risk of non-cancer death *is dependent on age at diagnosis*

Model(1) assumes that the increased risk of non-cancer death, expressed by parameter α, is independent of age at diagnosis. In order to assess the robustness of parameter estimations (especially α) with regard to variations of α according to age, we fitted Model(1) to data generated in breach of this assumption. We generated times to non-cancer death from expected survival probabilities given by

$${\textrm{S}}_{\textrm{E}}={\textrm{S}}^{\ast {\upalpha}_{\textrm{x}}},\textrm{with}\ {\upalpha}_{\textrm{x}}=\upalpha +{\textrm{b}}_{\upalpha}\left(\textrm{x}\hbox{-} \textrm{E}\left(\textrm{x}\right)\right)$$

where S* was all-cause survival from the population life table, α_x_ was the increased risk of non-cancer death as a function of age x at diagnosis; E(x) is the expected value of sampling age distribution (actually E(x) = 62.25 in all our samples), α was set at 1.2 and 2.0 (according to the considered scenario) and the slope coefficient b_α_ was set to provide a reasonable range of values and vary between − 0.08 and 0.05 (i.e. 5% per year of age). Model(1) would of course estimate a single α parameter common to all ages. Given the linear relationship, we have for each sample E(α_x_) = α.

#### The increased risk of non-cancer death varies randomly among patients

Model(1) assumes that the increased risk of non-cancer death, expressed by parameter α, acts as a fixed effect. We investigated the behaviour of Model(1) when applied to data generated with an increased risk of non-cancer death randomly assigned to the simulated cases. Log(α) was considered uniformly distributed around the overall value, and with increasing ratios of maximum to minimum values from 2 to 4.

## Results

### Performances of models when all assumptions were valid

The performance indicators of Model(1) are reported in Table 1 for estimation methods for both grouped and individual data. Statistics on all six parameters considered are shown. Parameters β and δ represent changes in the probability of cure and of cancer survival in the uncured due to a 15-year difference in age at diagnosis. Estimates of α were always very close to the true underlying values, with an absolute bias (AB) ranging from − 0.006 to 0.006 and a relative bias always lower than 0.6%. The estimates of π_60_ were also very close to their underlying values. The AB was low for all of the other parameters, for both scenarios, and for both estimation methods. It ranged between − 0.011 (β estimate, “Breast” scenario) and + 0.004 (β estimate, “Lung” scenario). The standard deviation of α estimates was directly related to the true value of the parameter. Their coefficient of variation (standard deviation divided by the mean, not shown) ranged between 6 and 15%. The standard deviation of π_60_ estimates was considerably lower than those of α, with the coefficient of variation of estimates always within the range 4 to 6%, thereby resulting in higher precision of the estimates compared with α. The standard deviation of the estimates was also generally low for λ and γ , but it was higher for the two trend parameters β and δ. The standard deviation for estimates based on individual data was in general slightly lower than that for grouped data. The coverage estimated was almost always slightly lower but close to the nominal value of 95% for both scenarios, both methods, and all parameters. One partial exception was the coverage of λ estimates for “Lung”, which ranged from 89 to 95%.

Table 1 shows no strong advantage of estimates based on individual data with respect to those based on grouped data, which consumed far less time and computer power. The following Tables present only results from the latter method, but the robustness analysis for individual data can be found in Supplementary Material Table 2.

The main performance indicators of Model(1), compared with the conventional cure model, are presented in Table 2. The conventional model gave unbiased estimates for all parameters when α = 1, with precision and coverage similar to those obtained from Model(1), but progressively more biased estimates of the parameters as the underlying value of α departed from 1.0. This is one more reason in favour of the systematic use of the full Model(1).

**Table 2:** Performance indicators of conventional model and Model(1) using Maximum likelihood estimation on grouped data by different α

Scenario	Method	true value α	AB						SD						CVR					
			α	π_**60**_	λ	γ	β	δ	α	π_**60**_	λ	γ	β	δ	α	π_**60**_	λ	γ	β	δ
BREAST^a^	conventional	2		− 0.281	− 0.042	0.011	−0.911	− 0.012		0.046	0.005	0.029	0.097	0.100		0%	0%	96%	0%	95%
	Model(1)	2	−0.005	−0.004	0.000	0.002	−0.011	−0.005	0.115	0.043	0.012	0.048	0.199	0.157	93%	93%	93%	95%	93%	94%
	conventional	1.5		−0.173	−0.030	− 0.028	−0.627	0.140		0.036	0.006	0.033	0.093	0.092		0%	0%	88%	0%	64%
	Model(1)	1.5	−0.001	−0.003	0.000	0.002	−0.007	−0.004	0.099	0.038	0.011	0.046	0.184	0.146	94%	93%	95%	95%	94%	94%
	conventional	1.2		−0.075	− 0.016	− 0.029	− 0.314	0.142		0.027	0.006	0.037	0.104	0.093		8%	30%	89%	18%	71%
	Model(1)	1.2	0.000	−0.003	0.000	0.002	−0.007	− 0.003	0.090	0.036	0.010	0.045	0.174	0.139	93%	94%	96%	96%	94%	94%
	conventional	1		−0.001	0.000	0.000	−0.002	0.000		0.018	0.007	0.040	0.097	0.106		95%	95%	95%	95%	94%
	Model(1)	1	−0.001	−0.003	0.000	0.002	−0.009	−0.001	0.084	0.034	0.010	0.044	0.169	0.134	93%	94%	95%	96%	94%	93%
	conventional	0.8		0.058	0.017	0.048	0.299	−0.221		0.011	0.008	0.042	0.084	0.106		2%	40%	79%	4%	41%
	Model(1)	0.8	−0.001	− 0.002	0.000	0.002	−0.007	−0.002	0.077	0.033	0.009	0.043	0.161	0.129	94%	92%	95%	95%	93%	93%
LUNG^b^	conventional	2		−0.010	− 0.012	− 0.032	− 0.131	0.017		0.004	0.013	0.014	0.061	0.023		22%	85%	35%	45%	86%
	Model(1)	2	−0.006	0.002	− 0.009	− 0.001	0.003	0.003	0.206	0.004	0.014	0.015	0.065	0.023	96%	92%	89%	95%	95%	95%
	conventional	1.5		−0.004	−0.011	− 0.016	− 0.061	0.011		0.004	0.013	0.014	0.059	0.023		84%	88%	76%	84%	92%
	Model(1)	1.5	− 0.004	0.002	− 0.009	− 0.001	0.003	0.003	0.165	0.004	0.013	0.014	0.063	0.023	96%	92%	89%	94%	95%	94%
	conventional	1.2		0.000	− 0.010	− 0.007	− 0.022	0.006		0.004	0.013	0.014	0.058	0.023		96%	89%	91%	95%	93%
	Model(1)	1.2	0.000	0.002	−0.009	0.000	0.004	0.003	0.149	0.004	0.013	0.014	0.062	0.023	95%	92%	90%	94%	94%	93%
	conventional	1		0.002	− 0.009	0.000	0.002	0.002		0.004	0.015	0.015	0.061	0.026		92%	98%	98%	95%	97%
	Model(1)	1	0.001	0.002	−0.009	0.000	0.004	0.003	0.132	0.004	0.013	0.014	0.061	0.023	96%	91%	90%	94%	94%	94%
	conventional	0.8		0.005	−0.008	0.006	0.028	0.000		0.004	0.013	0.013	0.057	0.022		75%	90%	92%	92%	94%
	Model(1)	0.8	−0.002	0.002	−0.009	0.000	0.003	0.003	0.121	0.004	0.013	0.014	0.060	0.022	95%	91%	90%	94%	94%	93%

Absolute Bias (AB) = Mean(estimates - true value); Standard Deviation (SD) = standard deviation over the set of 1000 estimates; Coverage (CVR) = the proportion of the time that the estimated 95% confidence intervals contained the true value. Estimated over 1000 simulation runs; Sample size 10,000; follow-up 15 years

^a^True values: π_60_ = 0.7, λ =0.1, γ =1.1, β = − 0.15 and δ = 0. ^b^True values: π_60_ = 0.1, λ =0.9, γ =0.8, β = − 0.75 and δ = − 0.3

Table 3 illustrates the behaviour of Model(1) with varying sample size N and length of potential follow-up. For the “Breast” scenario, decreasing the sample size and length of follow-up led to positive bias for α and a negative one for π. For the “Lung” scenario, π was generally estimated well, but both positive and negative AB of α estimates were obtained for 5 yrs. follow-up. This was also due to the very large standard errors. Estimates of α were more sensitive to decreasing sample size and length of follow-up than were those of π. Generally speaking, these results indicate that small sample sizes and short follow-up definitely led to unstable estimates. With at least 10 years of follow-up *N* ≥ 5000 were needed to obtain good model estimates and with at least 15 years of follow-up the model provides acceptable estimates with smaller sample size (*N* ≥ 1000). The latter is needed to provide the amount of information ensuring good estimate of α as well as the other involved parameters.

**Table 3:** Performance indicators using grouped data according to sample size and length of follow-up in years

Scenario	N	FUP	AB						SD						CVR					
			α	π	λ	γ	β	δ	α	π	λ	γ	β	δ	α	π	λ	γ	β	δ
BREAST^a^	10,000	5	0.094	−0.034	0.003	0.013	− 0.246	− 0.046	0.298	0.170	0.033	0.083	3.728	0.379	92%	84%	90%	92%	92%	92%
		10	0.009	−0.004	0.000	0.003	0.006	−0.012	0.144	0.048	0.013	0.052	0.229	0.179	94%	93%	95%	96%	94%	94%
		15	0.000	−0.003	0.000	0.002	−0.007	− 0.003	0.090	0.036	0.010	0.045	0.174	0.139	93%	94%	96%	96%	94%	94%
	5000	5	0.158	−0.049	0.007	0.024	−0.500	− 0.057	0.407	0.218	0.042	0.115	5.504	0.518	91%	78%	84%	90%	90%	90%
		10	0.022	−0.009	0.001	0.007	0.012	−0.029	0.207	0.079	0.020	0.074	0.408	0.258	92%	91%	94%	96%	93%	93%
		15	−0.003	−0.006	0.000	0.006	−0.013	−0.009	0.127	0.054	0.015	0.064	0.250	0.196	91%	90%	92%	95%	93%	92%
	2000	5	0.280	−0.056	0.017	0.067	−0.117	− 0.121	0.579	0.265	0.061	0.179	7.413	0.732	86%	69%	80%	91%	88%	86%
		10	0.053	−0.025	0.002	0.024	−0.132	− 0.112	0.320	0.146	0.031	0.122	3.012	0.436	89%	87%	93%	94%	89%	90%
		15	0.007	−0.020	−0.001	0.014	0.006	−0.038	0.193	0.096	0.024	0.106	0.470	0.308	91%	89%	92%	95%	91%	93%
	1000	5	0.325	−0.063	0.048	0.109	0.188	−0.041	0.874	0.287	0.494	0.250	9.474	0.990	81%	65%	80%	93%	86%	84%
		10	0.079	−0.048	0.003	0.042	−0.254	− 0.080	0.421	0.193	0.043	0.172	3.952	0.579	88%	82%	90%	94%	89%	90%
		15	0.003	−0.036	−0.001	0.028	−0.049	− 0.027	0.255	0.133	0.032	0.146	1.934	0.451	91%	87%	90%	95%	91%	89%
	500	5	0.308	−0.066	0.050	0.243	1.870	−0.089	1.208	0.316	0.202	0.469	15.490	1.307	76%	58%	79%	90%	88%	82%
		10	0.136	−0.059	0.011	0.104	0.195	−0.195	0.558	0.240	0.093	0.271	9.083	0.847	86%	74%	85%	92%	88%	85%
		15	0.036	−0.049	0.001	0.080	−0.108	− 0.128	0.347	0.193	0.049	0.236	6.109	0.667	89%	79%	86%	92%	89%	87%
LUNG^b^	10,000	5	−0.169	0.002	−0.008	0.000	−0.010	0.003	1.595	0.012	0.017	0.021	0.138	0.030	95%	95%	92%	96%	96%	94%
		10	−0.016	0.002	−0.009	−0.001	0.002	0.003	0.386	0.005	0.014	0.016	0.074	0.024	95%	92%	90%	94%	95%	95%
		15	−0.006	0.002	−0.009	−0.001	0.003	0.003	0.206	0.004	0.014	0.015	0.065	0.023	96%	92%	89%	95%	95%	95%
	5000	5	−0.130	0.002	−0.008	0.001	−0.004	−0.001	2.341	0.017	0.023	0.029	0.202	0.043	94%	94%	93%	95%	93%	95%
		10	−0.028	0.002	− 0.009	0.001	0.005	0.001	0.555	0.008	0.020	0.022	0.110	0.034	95%	93%	92%	95%	94%	94%
		15	0.013	0.003	−0.009	0.001	0.009	0.001	0.305	0.007	0.019	0.021	0.096	0.032	94%	93%	92%	95%	94%	95%
	2000	5	−0.079	0.002	−0.007	0.005	−0.006	−0.004	3.792	0.027	0.036	0.050	0.399	0.070	94%	92%	95%	95%	94%	95%
		10	0.039	0.003	−0.008	0.003	0.006	0.004	0.927	0.013	0.031	0.037	0.170	0.053	94%	95%	93%	94%	95%	95%
		15	0.009	0.002	−0.009	0.001	0.003	0.005	0.502	0.011	0.031	0.035	0.148	0.051	93%	94%	93%	95%	95%	95%
	1000	5	0.419	0.005	−0.003	0.012	−0.027	− 0.008	5.265	0.038	0.054	0.070	1.379	0.104	92%	92%	95%	96%	93%	95%
		10	0.072	0.003	−0.007	0.009	0.003	0.003	1.246	0.018	0.044	0.053	0.242	0.075	96%	94%	93%	94%	97%	95%
		15	0.006	0.002	−0.008	0.005	−0.002	0.004	0.705	0.015	0.043	0.049	0.212	0.072	93%	93%	94%	95%	96%	95%
	500	5	1.986	0.012	0.004	0.025	0.045	−0.031	7.705	0.056	0.080	0.108	4.088	0.166	90%	89%	95%	94%	90%	93%
		10	0.462	0.006	−0.005	0.019	0.050	−0.011	1.777	0.025	0.063	0.073	0.352	0.112	98%	95%	95%	95%	95%	94%
		15	0.116	0.003	−0.009	0.012	0.019	−0.007	1.003	0.021	0.062	0.068	0.306	0.106	93%	96%	95%	95%	95%	94%

Absolute Bias (AB) = Mean (estimates - true value); Standard Deviation (SD) = standard deviation over the set of 1000 estimates; Coverage (CVR) = the proportion of the time that the estimated 95% confidence intervals contained the true value. Estimated over 1000 simulation runs each composed by different sample size and follow-up lenght in years (FUP)

^a^True values: π_60_ = 0.7, λ =0.1, γ =1.1, β = − 0.15 and δ =0

^b^True values: π_60_ = 0.1, λ =0.9, γ =0.8, β = − 0.75 and δ = − 0.3

Obviously the ability of the Model(1) to produce acceptable results with similar sample size and length of follow-up varied also according to the amount of deaths, and these general conclusions can be relaxed. Indeed, a cohort of 500 cases will produce acceptable estimates when lethal cancer sites and long follow-up (15 years) are studied.

Within these conditions, the SD of α and π were roughly inversely proportional to the square root of sample size and increased with decreasing lengths of follow-up. The same conclusion was drawn when individual data were used (Supplementary Material Table 3 ).

### Robustness analysis

All the previous results were obtained for samples generated according to the model assumptions. In the following paragraph, we report the performances of Model(1) when some of these assumptions were violated by the data generation algorithm.

#### The times to cancer death of uncured patients did not follow a Weibull distribution

The different shapes of the hazard and cumulative survival functions obtained by varying θ under the two considered scenarios are plotted in Supplementary Material Fig 1. The two dotted lines are those with minimum θ = 0.25, showing a high initial risk, followed by a minimum value and by an increasing risk, and those with the maximum θ = 4, showing an opposite pattern, increasing at the beginning, but with a decreasing trend in the long term. The reference function with θ = 1, corresponding to the Weibull distribution defined for each scenario, is represented by the black lines. Figure 1 in the supplementary material shows that values of θ not equal to 1 change both the pattern and the level of the hazard. As a consequence, the other survival parameters λ, γ, and δ, also have to change in order to fit the same dataset.

Performance indicators of estimates are summarized in Table 4 for all parameters apart from λ and γ, for which we did not have reference true values. Estimates of almost all parameters presented an increasing pattern for increasing values of θ except for α for “Breast” and δ. In order to balance the bias of β the AB of δ presented a decreasing pattern for increasing values of θ in both scenarios. The estimates of parameter α under the “Lung” scenario were the most sensitive to the value of θ, increasing from 1.89 to 2.81 (AB − 0.106 to 0.815) around the true value of 2.0. The estimates of the other parameters π_60_ , and β increased respectively from 10 to 13% and from − 0.75 to − 0.59.

**Table 4:** Performance indicators of Model(1) when applied to data generated with exponential-Weibull distribution of survival for uncured patients

Scenario	θ	AB				SD				CVR			
		α	π_**60**_	β	δ	α	π_**60**_	β	δ	α	π_**60**_	β	δ
BREAST^a^	0.25	− 0.021	− 0.058	− 0.069	0.038	0.061	0.057	0.152	0.125	92%	96%	95%	92%
	0.5	−0.024	− 0.045	− 0.069	0.040	0.072	0.040	0.156	0.126	93%	93%	94%	93%
	0.625	−0.021	−0.035	−0.058	0.031	0.078	0.038	0.160	0.129	94%	95%	94%	94%
	0.8	−0.011	−0.019	−0.034	0.015	0.084	0.037	0.169	0.134	94%	96%	94%	93%
	1.25	0.010	0.013	0.018	−0.020	0.095	0.036	0.184	0.144	92%	87%	93%	93%
	1.6	0.009	0.029	0.027	−0.027	0.096	0.037	0.190	0.148	93%	80%	93%	93%
	2	−0.003	0.038	0.017	−0.026	0.092	0.038	0.193	0.150	94%	73%	94%	94%
	4	−0.014	0.082	0.018	−0.026	0.062	0.047	0.315	0.227	96%	35%	97%	94%
LUNG^b^	0.25	−0.106	− 0.001	0.003	0.002	0.185	0.004	0.061	0.027	91%	94%	95%	94%
	0.5	−0.105	−0.001	− 0.003	0.005	0.193	0.004	0.063	0.024	91%	95%	95%	95%
	0.625	−0.092	0.000	− 0.005	0.005	0.198	0.004	0.064	0.023	93%	96%	95%	95%
	0.8	−0.056	0.001	−0.002	0.004	0.201	0.004	0.064	0.023	95%	94%	96%	95%
	1.25	0.072	0.005	0.014	0.001	0.213	0.005	0.065	0.023	95%	85%	95%	94%
	1.6	0.194	0.008	0.032	−0.003	0.225	0.005	0.066	0.023	87%	65%	92%	94%
	2	0.340	0.012	0.056	−0.007	0.239	0.005	0.066	0.023	70%	36%	86%	93%
	4	0.815	0.028	0.158	−0.029	0.270	0.006	0.071	0.027	7%	0%	36%	76%

Absolute Bias (AB) = Mean(estimates - true value); Standard Deviation (SD) = standard deviation over the set of 1000 estimates; Coverage (CVR) = the proportion of the time that the estimated 95% confidence intervals contained the true value

Estimated over 1000 simulation runs; Sample size 10,000; follow-up 15 years

The performance indicators for λ and γ are omitted from the table, even if estimated, due to impossibility to compare the estimated values with the fixed true values used in the simulation process

^a^True values: α = 1.2, π_60_,=0.7, λ = 0.1, γ = 1.1, β = − 0.15 and δ = 0

^b^True values: α = 2, π_60_,=0.1, λ = 0.9, γ = 0.8, β = − 0.75 and δ = − 0.3.

As for the “Breast” scenario, the estimates of all three parameters were more stable and closer to their true values. The AB for α (true value = 1.2) ranged from − 0.024 to 0.01, those of π_60_ (true value = 70%) ranged from-0.058 to 0.082, while those of β (true value = − 0.15) ranged from − 0.069 to 0.018.

#### The increased risk of non-cancer death is dependent on age at diagnosis

In Table 5, we report the performance indicators of Model(1) when data were generated under a linear trend of α with age. The column headed b_α_ shows the linear coefficient of the age trend and in the following column is presented the range: the minimum and maximum values of α at ages 40 and 75, respectively. The limit coefficient values were taken so as not to have implausible protective levels of α for the extreme age classes.

**Table 5:** Performance indicators of Model(1) in presence of variation of α according to age at diagnosis

Scenario	b_**α**_	Range		AB						SD						CVR					
		α_**40**_	α_**75**_	α	π_**60**_	λ	γ	β	δ	α	π_**60**_	λ	γ	β	δ	α	π_**60**_	λ	γ	β	δ
BREAST^a^	−0.02	1.65	0.95	− 0.271	− 0.083	− 0.017	− 0.038	− 0.040	−0.043	0.102	0.059	0.011	0.043	0.251	0.175	24%	75%	59%	82%	88%	90%
	−0.01	1.43	1.08	−0.129	− 0.037	− 0.008	− 0.017	0.070	− 0.104	0.098	0.047	0.011	0.045	0.210	0.163	72%	93%	85%	92%	89%	89%
	−0.005	1.31	1.14	−0.065	− 0.020	− 0.004	− 0.008	0.109	− 0.129	0.095	0.042	0.011	0.045	0.192	0.155	90%	95%	92%	94%	86%	86%
	0.001	1.18	1.21	0.012	0.000	0.001	0.004	0.001	−0.008	0.090	0.035	0.010	0.045	0.172	0.138	93%	93%	96%	95%	93%	94%
	0.002	1.16	1.23	0.025	0.003	0.002	0.006	0.008	−0.013	0.089	0.034	0.010	0.045	0.169	0.137	93%	92%	96%	95%	94%	94%
	0.005	1.09	1.26	0.061	0.011	0.004	0.011	0.028	−0.026	0.088	0.032	0.010	0.045	0.163	0.134	89%	89%	95%	95%	93%	94%
	0.01	0.98	1.33	0.120	0.023	0.007	0.020	0.058	−0.047	0.085	0.029	0.010	0.046	0.152	0.130	72%	81%	92%	94%	92%	94%
LUNG^b^	−0.08	3.80	1.00	−0.501	0.000	−0.013	− 0.008	0.030	− 0.006	0.174	0.004	0.013	0.015	0.064	0.023	20%	95%	84%	91%	93%	94%
	−0.04	2.90	1.50	−0.239	0.002	−0.011	− 0.004	0.017	− 0.001	0.192	0.004	0.014	0.015	0.065	0.023	75%	94%	87%	93%	94%	94%
	−0.02	2.45	1.75	−0.118	0.002	−0.010	− 0.002	0.010	0.001	0.199	0.004	0.014	0.015	0.065	0.023	90%	93%	89%	94%	95%	95%
	0.01	1.78	2.13	0.046	0.003	−0.009	0.000	0.000	0.004	0.208	0.004	0.014	0.015	0.065	0.023	97%	91%	89%	95%	95%	95%
	0.02	1.55	2.25	0.098	0.003	−0.008	0.001	−0.004	0.005	0.209	0.004	0.014	0.015	0.065	0.023	95%	92%	90%	95%	95%	95%
	0.04	1.10	2.50	0.194	0.003	−0.007	0.002	−0.012	0.007	0.218	0.004	0.014	0.015	0.066	0.023	88%	91%	91%	95%	94%	94%
	0.05	0.88	2.63	0.238	0.003	−0.007	0.003	− 0.016	0.008	0.221	0.004	0.014	0.015	0.066	0.023	84%	90%	91%	94%	94%	93%

Absolute Bias (AB) = Mean(estimates - true value); Standard Deviation (SD) = standard deviation over the set of 1000 estimates; Coverage (CVR) = the proportion of the time that the estimated 95% confidence intervals contained the true value

Estimated over 1000 simulation runs; Sample size 10,000; follow-up 15 years

^a^True values: α = 1.2, π_60_ = 0.7, λ = 0.1, γ = 1.1, β = − 0.15 and δ = 0

^b^True values: α = 2, π_60_ = 0.1, λ = 0.9, γ = 0.8, β = − 0.75 and δ = − 0.3

The AB in estimating α was higher for “Breast”, with higher survival, than for the “Lung” scenario, for similar b_α_. It remained smaller than 0.1 and greater than − 0.1 when the rate of increase of α was between − 0.005 and 0.005 per year of age for “Breast” and between 0.01 and 0.02 for “Lung”. In both scenarios, the AB was roughly proportional to the slope b_α_. Thus, α estimates tended to their true values at older ages, for which the parameter has the higher impact due to the higher mortality for other causes. The other parameters were similarly biased by a breach of the α-age independency assumption for the “Breast” scenario and a negative slope, and were hardly affected in all other cases.

#### The increased risk of non-cancer death varied randomly among patients

Table 6 shows what happened when α was generated as a random effect. Mean estimates of α, assumed in Model(1) as a fixed effect covariate, slightly decreased with increasing random variability of the underlying parameter. The AB was non-negligible for α in both scenarios when the range of the max/min ratio became greater than 2 (− 0.11 for “Breast” and − 0.16 for “Lung” when the widest range of variability was considered). AB always remained closer to zero and by less than 0.05 for all other parameters. Estimates of β and δ were sensitive to the variability of α only for the “Breast” scenario, where showed higher AB but maintained a coverage close to 95%. In all the other cases, the estimates of π, λ, and γ parameters were only marginally affected by underlying α variability, and remained close to their true values.

**Table 6:** Performance indicators of Model(1) in presence of data generated with α randomly assigned among patients

	α range	AB						SD						CVD					
		α	π	λ	γ	β	δ	α	π	λ	γ	β	δ	α	π	λ	γ	β	δ
BREAST^a^	0.832–1.664	− 0.024	− 0.006	− 0.001	0.003	− 0.019	− 0.001	0.091	0.036	0.010	0.044	0.165	0.132	95%	94%	94%	94%	95%	95%
	0.659–1.978	− 0.066	− 0.013	− 0.002	− 0.004	− 0.042	0.022	0.089	0.036	0.010	0.044	0.160	0.126	88%	96%	94%	95%	95%	95%
	0.555–2.218	−0.106	− 0.019	− 0.002	− 0.003	− 0.039	0.019	0.088	0.036	0.010	0.043	0.155	0.120	77%	95%	91%	95%	94%	96%
LUNG^b^	1.386–2.773	−0.042	0.002	−0.009	− 0.001	0.002	0.003	0.204	0.004	0.014	0.015	0.064	0.022	95%	93%	89%	96%	95%	96%
	1.099–3.296	−0.108	0.001	− 0.010	− 0.002	− 0.002	0.003	0.201	0.004	0.014	0.015	0.064	0.022	91%	95%	89%	96%	96%	96%
	0.924–3.697	−0.165	0.001	−0.010	− 0.002	− 0.006	0.003	0.196	0.004	0.014	0.015	0.063	0.022	86%	96%	89%	95%	96%	96%

Absolute Bias (AB) = Mean(estimates - true value); Standard Deviation (SD) = standard deviation over the set of 1000 estimates; Coverage (CVR) = the proportion of the time that the estimated 95% confidence intervals contained the true value

Estimated over 1000 simulation runs; Sample size 10,000; follow-up 15 years

^a^True values: α = 1.2, π_60_ = 0.7, λ = 0.1, γ = 1.1, β = − 0.15 and δ = 0

^b^True values: α = 2, π_60_ = 0.1, λ = 0.9, γ = 0.8, β = − 0.75 and δ = − 0.3

### Application to real-life FRANCIM colon cancer data

As an example, this method was applied to survival data of colon cancer patients recorded by FRANCIM (the French network of cancer registries). FRANCIM data are checked for quality and completeness every 4 years by an independent audit committee (Comité d’Évaluation des Registres). Life tables were provided by the National Statistics Institute (INSEE). All colon cancers diagnosed in 1995–2009 in patients aged 40–74 were included (*N* = 15,717 in men and *N* = 10,942 in women). The relative survival and the cure fraction were estimated, separately for each sex, using Model(1) and using the conventional model without the α parameter (Table 7). The cure assumption was already checked for these data [21].

**Table 7:** Real data application: (A) Parameters’ estimation (standard errors) and (B) net survival and proportion of cured

**A)**												
	**Model(1)**						**Conventional mixture cure model**					
	**α**	**π**_**60**_	**λ**	**γ**	**β**	**δ**	**α**	**π**_**60**_	**λ**	**γ**	**β**	**δ**
**Men**	1.29 (0.066)	57.4 (0.049)	0.42 (0.014)	0.88 (0.023)	−0.1 (0.047)	−0.16 (0.046)	1	52.4 (0.033)	0.39 (0.018)	0.83 (0.042)	−0.22 (0.042)	−0.08 (0.095)
**Women**	1.32 (0.084)	60.7 (0.036)	0.4 (0.012)	1 (0.026)	−0.04 (0.04)	−0.25 (0.045)	1	58.3 (0.028)	0.38 (0.021)	0.94 (0.038)	−0.11 (0.041)	−0.18 (0.334)
**B)**												
**Gender**	**Age at diagnosis**	**Model(1)**					**Conventional mixture cure model**					
		**1-y. NS**	**5-y. NS**	**10-y. NS**	**15-y. NS**	**π**	**1-y. NS**	**5-y. NS**	**10-y. NS**	**15-y. NS**	**π**	
**Men**	40	89%	70%	63%	61%	60%	88%	71%	64%	61%	60%	
	50	87%	67%	61%	60%	59%	87%	67%	60%	57%	56%	
	60	85%	65%	59%	58%	57%	85%	63%	56%	54%	52%	
	70	83%	62%	57%	56%	56%	83%	60%	52%	50%	49%	
**Women**	40	91%	71%	64%	63%	62%	90%	71%	65%	63%	62%	
	50	89%	69%	63%	62%	61%	88%	69%	62%	61%	60%	
	60	87%	66%	61%	61%	61%	87%	66%	60%	59%	58%	
	70	85%	64%	60%	60%	60%	85%	63%	57%	57%	56%	

Colon cancer data in France

*NS* net survival, *π* proportion of cured

For both sexes, we estimated α ≅ 1.3, with confidence intervals not including 1, therefore supporting the hypothesis of increased non-cancer mortality in colon cancer patients. The differences between the cure fraction estimates from Model(1) and those from the conventional model were greater for males (57% vs. 52% at age 60) than for females (61% vs. 58%) due to the higher mortality rates for other causes in the male population. Model(1) and the conventional model also provided different estimates for survival by age. For example, 10-yr relative survival in males decreased from 63% at age 40 to 57% at age 70 with Model(1), and from 64 to 52%, respectively, with the conventional model. Since non-cancer mortality increases steeply with age, not including alpha in the model led to a portion of the age trend in mortality being attributed to cancer rather than to other causes. Not taking into consideration the cancer patients’ increased risk of non-cancer death led to bias in the colon cancer net survival estimators produced so far.

## Discussion

This simulation study, which showed very good performances, validated the mixture cure model for relative survival estimation presented here. The model includes a coefficient that reflects the risk of dying from causes other than cancer in cancer patients as compared with the risk of all-cause death in the general population. In the rationale of this study this coefficient expressed an increased risk of non-cancer death for cancer patients (α > 1) but we also validated the model for α =0.8, as we could not exclude the possibility that, for some specific cancers, the risk of non-cancer death in the patients can be smaller than the risk of death in the general population.

The model was tested in the ideal situation, where it exactly mirrored the pattern of data to which it was applied. In these simulations, we considered two extreme scenarios in terms of cure fraction, survival time of uncured patients, and age trends of both quantities, reflecting the survival pattern of lung and breast cancer. We considered linear age effects in order to reduce the number of parameters to be presented and because other analyses with categorical age effects provided similar results. We applied the model to both individual data and grouped data, the latter in the form of life tables stratified by age at diagnosis. When Model(1) assumptions were verified, the two methods showed comparable performances and provided unbiased estimates of the parameters, and coverage close to its nominal value of 95%. Standard deviations of estimates of α and π_60_ over the 1000 simulation runs using individual data were only slightly lower than those using grouped data. When there was an increased risk of non-cancer death, the estimates generated by the conventional model carried a risk of being severely biased. When there was no increased risk, Model(1) generated the same estimates as the conventional model. A sample size of 5000 cases with more than 10 years of follow-up were sufficient to obtain reliable estimates for all parameters in most scenarios.

Good model specification is a requirement of every modelling application, but seldom can it be completely achieved or checked. In the second part of this work, we studied the robustness of model estimates when the underlying assumptions were not in line with the data, more specifically: when relative survival in uncured patients did not follow a Weibull distribution; when the increased risk of non-cancer death was dependent on age at diagnosis; when the increased risk of non-cancer death was not a fixed effect but randomly varied within the cohort of patients. The individual data approach provided (Supplementary material Table 2) severely biased estimates in some extreme scenarios, in particular for the low survival “Lung” scenario with exponential Weibull parameters θ = 0.25. This was perhaps due to the extremely high probability density generated by this distribution in very short times (say, 1–2 months) after diagnosis, making the contribution of observations with longer follow-up time negligible. The analysis of grouped data was also faster and is suitable to overcome problems of data access, problems due to personal data protection regulations, which research teams are increasingly facing. The grouped data approach was therefore chosen to obtain a more detailed presentation of results.

Each of the mis-specification situations considered could be accounted for by modifying the basic model, i.e. by considering other survival distributions, or including age dependence, or random effect covariates. This could be the subject of future developments on corrected cure models. Nevertheless, investigating the robustness of a parsimonious model can help in finding a compromise between a lack of fit and over-parametrization.

The strength of the corrected model studied here lies in the fact that an overall risk of death from other causes can be estimated without needing to know the causal factors and their distribution in the patient set and in the general population. Such information is seldom available in population-based studies. On the other hand, the model assumes the increased risk of death in patients to act as a multiplicative relative risk with respect to mortality in the general population. The multiplicative relative risk model has, however, shown its plausibility during its broad and long use in epidemiological research. Its appropriateness in the specific field of cancer survivorship can be ascertained through an extensive application to epidemiological data on various cancers and in different populations.

The model is based on the plausible assumption that patients’ cancer mortality is not indefinitely increasing after some years since diagnosis, differently from the increasing age trend of other causes mortality. This makes in principle possible to disentangle the two risks. The model can be fitted even if the excess death remains constant after a long time and in this context the functional form has to be found that best describes the analysed data. Actually, we found a good behaviour of the model also in scenarios assuming persisting long-term cancer mortality, as expressed by γ parameter = 1.1 (Breast scenarios).

In order to test the model behaviour in a critical situation, it was also applied to data generated with negligible or zero proportion of cure and, in addition, with persisting long-term cancer mortality. Actually, we found that the corrected cure model provided biased estimates for α and for the cure fraction (Supplementary material Table 1). Such bias was however attributable to the logistic transformation. Indeed, when applying a linear age effect on the cure fraction, all estimates were unbiased (Supplementary material Table 1). Finally, we compared the estimates provided by the full model and those from a model obtained by retaining the α parameter but with no cure assumption (Supplementary material Table 1). The latter provided biased estimates only when the true cure fraction was higher than 5%. It can be suggested that, when in the applications the full corrected model gives an estimated cure fraction of less than 10%, the no-cure model, with the inclusion of α parameter, should be also applied for comparison. Moreover the model with linear effect of age on the cure fraction can be considered in the case of non-negative estimate of the cure fraction.

This work has several limitations. We only tested the model with the Weibull distribution assumption for survival of the uncured patients. The Weibull is a very popular survival distribution generally providing a good fit of cancer data [25] but other choices are possible as the lognormal [26], the loglogistic [27], or the flexible regression spline-generated [28] distributions. We have no reason to believe that any of these distributions would give different results in the case of well-specified model, when exactly the same distribution is used to generate and to fit the data. However, their behaviour may change when some of the assumptions are not verified, so the robustness analysis could be affected by the specific function considered.

Reliable cause-of-death data from population-based sources can be used to test the distribution assumptions considered. Cause of death was not available in the data of our application. In the future this information will be standardized and included in the basic dataset of cancer registries.

Age at diagnosis was the only covariate included in the model. Other variables available from population-based data, as sex, period of diagnosis or, sometimes, stage and treatment, can be included without substantially modifying the model structure.

A linear effect of age, as considered in the model, can be accepted for many cancers, but can be unrealistic for others. Breast and prostatic cancers have for instance a higher death risk for the young and the old with respect to the middle age patients. We choose the linear link to simplify the proof of concept, mainly focused on the alpha parameter, without great loss of generality. Second or higher order polynomials can be used to account for different cancer mortality pattern such as U-shaped.

Future methodological development can address the testing of other scenarios, designed to mirror a wider variety of cancers, exploring different survival distributions for the uncured and separately modelling the parameters in the distribution function, that is, the scale (λ) and shape (γ) parameters in the case of the Weibull distribution.

Previous applications of the same model to real data [17, 18] or of survival models not focused on cure [13–15] have shown that an increased patients’ mortality risk due to other causes exists for several cancers. Reasons for different ‘other causes of death’ risk in patients with respect to the general population may differ. They range from causes not generated by the cancer such as smoking, genetic and other lifestyle risk factors common to cancer and other diseases, as well as to deprivation and other socio-economic factors, to factors indirectly caused by the diagnosed cancer such as side effects of cancer treatment [29, 30]. We cannot separate these two components without detailed treatment data from clinical databases. However, our intention was to capture cure of the cancer itself, implying no further risk of progression and death due to the diagnosed cancer and following the definition proposed by Haupt in 2007 “cure from the original cancer regardless of any potential for, or presence of, remaining disabilities or side effects of treatment” [31]. Following this definition we distinguish from patients that will die for progression or relapse of the diagnosed cancer and those who will die from other causes related (e.g. adverse effects of treatments) or not (e.g. other disease) with the cancer [32] .

This increased risk of death is greater in patients aged 65 or more. By applying our model to colon cancer data, we showed that not acknowledging an increased risk of death led to substantial bias in relative survival estimations and cure fractions. Even a moderately increased risk of 1.3 can make a greater difference than, for example, the one induced by different choices of the relative survival estimator, an issue that has raised considerable debate within the biostatistics community [33–35].

Concerning applications, the model provides a new indicator in cancer patients: the relative risk of death due to other causes. Estimating and accounting for variations in this indicator over time, in different populations, or according to stage or treatment could potentially improve the validity of survival comparisons. Moreover, we believe that providing correct estimates of cure fractions and of survival in uncured patients is important in that it could be used to improve the planning of health services for cancer survivors, particularly when a large increase in long-term cancer prevalence occurs or is expected. For instance, the estimated increased risk of dying of 1.3% in patients cured for colon cancer indicates that long-term clinical follow-up should focus more on preventing or treating the long-term effects of surgery and chemotherapies and addressing risk factors for cancer that are shared with other chronic diseases. To be more effective in preventing or reducing the increased risk, the same services that provide the cancer clinical follow-up have to take care of the conditions responsible for the increased risk estimated by this study, particularly in the elderly.

Correct estimates of time to cure, derived from the cure model parameters, also have practical implications in the legislation addressing the “Right to be Forgotten” of cancer patients https://ecpc.org/policy/the-right-to-be-forgotten-a-new-research-project. Such legislation has been introduced in France, Belgium, Luxembourg and the Netherlands, with efforts being made across Europe to establish similar approaches. Finally, a more accurate balance between the risk of death due to cancer and that due to other, often more effectively controlled, causes can improve patients’ awareness and quality of life.

## Conclusion

The present analysis supports the use of the corrected mixture cure model including the increased risk of non-cancer death, to provide better estimates of indicators based on cancer survival, which are important to public health decision-making and should improve patients’ awareness of their health status and facilitate their return to normal life.

## Supplementary Information

**Additional file 1: Supplemetary file Fig. 1.** Probability density according to different θ parameters representing different distribution of the uncured function.Two figures representing Lung and Breast scenario. **Supplemetary file Table 1.** Performance indicators using grouped data of Model(1) in situations when there was very small or no cure at all and a persisting long-term cancer mortality (g=1). The Model(1) was used with a logistic age effect on cured (as proposed all along the manuscript), a linear age effect on cured and without cure. 1,000 estimates each composed by 10,000 cases and 15 years of follow-up. **Supplementary file Table 2.** Robustness analysis using Maximum likelihood on individual data. a) The times to cancer death of uncured patients do not follow a Weibull distribution; b) The extra non-cancer death risk is dependent of age at diagnosis; c) The extra non-cancer death risk randomly varies among the patients. **Supplementary file Table 3.** Performance indicators for a and p using individual data according to sample size and length of follow-up. 1,000 estimates each composed by different sample size and follow-up length in years.

## Data Availability

*Data availability statement* The datasets used and/or analysed during the current study are available from the corresponding author upon reasonable request. The French colon cancer data that support the findings of this study are available from Francim but restrictions apply to the availability of these data, which were used under license for the current study, and so are not publicly available. Data are however available from the authors upon reasonable request and with the permission of Francim. *Code availability* Details of the Stata code and R-Package curesurv used to fit the proposed corrected mixture cue model are available in the Github and Zenovo repository. The repository is called *curesurvTools* (https://github.com/LauraBotta/curesurvTools and https://zenodo.org/record/7567886). Please cite this material as in reference [36]. The code including the unweighted least square estimation of Model(1) parameters, is available from the first author. The results for the latter method are close but a jackknife technique was required to obtain good coverage.

## Abbreviations

SEER: Surveillance, Epidemiology, and End Results Program
RS: Relative survival
FRANCIM: French network of cancer registries
INSEE: French National Statistics Institute
AB: Absolute Bias
SD: Standard Deviation
CVR: Coverage
P_AC_: Probabilities of administrative censoring
P_LF_: Probabilities of loss to follow-up
